# Liu-type pretest and shrinkage estimation for the conditional autoregressive model

**DOI:** 10.1371/journal.pone.0283339

**Published:** 2023-04-04

**Authors:** Marwan Al-Momani

**Affiliations:** Department of Mathematics, University of Sharjah, Sharjah, UAE; Semnan University, IRAN, ISLAMIC REPUBLIC OF

## Abstract

Spatial regression models have recently received a lot of attention in a variety of fields to address the spatial autocorrelation effect. One important class of spatial models is the Conditional Autoregressive (CA). Theses models have been widely used to analyze spatial data in various areas, as geography, epidemiology, disease surveillance, civilian planning, mapping of poorness signals and others. In this article, we propose the Liu-type pretest, shrinkage and positive shrinkages estimators for the large-scale effect parameter vector of the CA regression model. The set of the proposed estimators are evaluated analytically via their asymptotic bias, quadratic bias, the asymptotic quadratic risks, and numerically via their relative mean squared errors. Our results demonstrate that the proposed estimators are more efficient than Liu-type estimator. To conclude this paper, we apply the proposed estimators to the Boston housing prices data, and applied a bootstrapping technique to evaluate the estimators based on their mean squared prediction error.

## 1 Introduction

Data collected across geographical areas may show some dependencies in which closer observations are more similar than those farther apart. This behaviour can be modeled by incorporating a covariance structure into the traditional statistical models. One of these models is the spatial regression model, which assimilate different types of special dependencies. Applications of the spatial regression models have been growing up in different fields as ecology, epidemiology, disease mapping, public health, psychology, and others.

In the context of time series, Autoregressive models represent the error terms at time (*t*) as a linear function of the recent inherent errors. Similarly, autoregressive models in spatial framework model the data from a specific location, known as site, as a function of data from nearby locations, where a site is a physical location where the data is collected, and the conception of neighborhood between two sites is defined based on a specific distance or closeness metric. One important class of Spatial regression models is the Conditional Autoregressive model. The CA name is due to the possibility of writing the mean and the variance using conditional expectation form. The CA model has recently been extensively applied in a vast range of different areas. For example, but not limited to, Shen X. *et.al* [1] proposed CA model to analyze the heterogenous genetic effects among individuals which is considered as a random effect in their model. Pérez-Molina [2] modeled hierarchical relationships using multilevel models with random intercepts and a CA component to account for spatial effects. He demonstrated that such models are significantly improve housing price modeling. Tharmin S.A *et.al* [3] used Bayesian CA in mapping the relative risk of the spread of dengue fever disease in Makassar, Indonesia. They demonstrated that Makassar is still vulnerable to dengue fever. Qiang Z. *et.al* [4] proposed a Bayesian bivariate CA model to establish the links between crash frequencies and traffic attributes. Dibakar S. *et.al* [5] investigated the relationship between bicycle crash frequency and the factors that contribute to them at the census block group level in the state of Florida, USA, using the class of CA models within the hierarchical Bayesian framework. Ver Heof J.M. *et.al* [6] discussed six different types of practical ecological inferences that can be made using the CA and SA models. They compared the CA and simultaneous autoregressive (SA) models and demonstrated their evolution as well as their connection to partial correlations. Wang C. *et.al* [7] used spatial Poisson-lognormal with CA priors to investigate the impact of traffic congestion of road accidents. Kleinschmidt I. *et.al* [8] explored the spatial and temporal variation in small-area malaria incidence rates using CA models. Gelfand, A. E., and Vounatsou, P. [9] used multivariate CA models for the analysis of spatial data and there models to study the child growth and the spatial variation in HLA-B allele frequencies.

In classical Statistical inference, we use the sample data information (subjective information) to make inference about the unknown parameter(s). In Bayesian framework, we combine the non-sample information, known as Uncertain Prior Information(UPI) and the sample information to make the inference. The UPI can be obtained from different resources, for example, historical information about the parameter(s), or applying some selection methods used in regression analysis. In many cases, researchers have previous knowledge about some of the regression variables that will be used in their regression model, or may formulate a linear hypothesis of the form *H*_0_ : ***Hβ*** = ***h***, where ***β*** is a (*p* × 1) vector of regression coefficients, ***H*** is (*p*_2_ × *p*) known matrix of rank (*p*_2_ ≤ *p*), and ***h*** is a (*p*_2_ × 1) fixed vector of constants in ${\mathbb{R}}^{p_{2}}$. This restriction is a commonly used method in regular regression, experimental design, machine learning and other fields to produce a restricted model that can perform at least as well as the full model with all available predictors. It can also be considered as a variable selection technique in which the reduced model will be tested to investigate the importance of some variables in explanting the variation in the response variable, and to decide how really the model is useful in the prediction process. In our case, we use this hypothesis to produce a sub model with less number of predictors. Theoretically we assume such restriction to study the performance of the reduced model compared with the full one, and numerically, we force some of the coefficients to be zeros (not significant) to confirm our analytical results. In real life problems we can gain some knowledge about the important variables, eliminate redundancy of some variables, and figure out the multicolinearity issue using different techniques, as the AIC, BIC, best subset, penalization algorithms, and others. The correlation matrix among all variables including the responses will also a helpful tool to justify our restriction.

One of the oldest methods that combines the sample and the UPI is the pretest estimation. The pretest estimator combines the sample data model, known as full model, and the UPI model, known as the submodel, into the estimation process using a binary weights, and chooses the submodel estimator if the test statistics rejects the null hypothesis (*H*_0_) at a specific level of significance *α*, and the full model estimator otherwise. Later on, a new estimator that uses a smooth function of the test statistics is the shrinkage estimator. Further, an improved version of the shrinkage estimator, known as the positive shrinkage estimator was proposed. The three estimators have been discussed a lot in the literature under different settings. Al-Momani M. *et.al* [10] proposed the pretest, shrinkage and positive shrinkage estimators for the vector of regression coefficients of the marginal model with multinomial response, and showed the superiority of the positive shrinkage estimator over the classical generalized estimating equation (GEE). Al-Momani, M. and Dawod B.A. [11] used the idea of pretest, and the shrinkage estimation for the Autoregressive Conditionally Heteroscedastic (ARCH) model. They discovered that the positive shrinkage estimator outperformed the restricted, pretest, and shrinkage estimators regardless of the accuracy of the restriction provided by the linear hypothesis to check whether some of the coefficients of the ARCH model’s parameter vector are not significant is true or not. Li, Y and Jin, B [12] investigated the sparsity and homogeneity of regression coefficients using prior constraint information in their work and showed combining prior knowledge can increase the effectiveness of both sparsity and homogeneity identification. Arumairajan, S. [13] proposed a stochastic restricted Liu estimator that is almost unbiased by combining modified nearly unbiased Liu estimator and mixed estimator when multicollinearity is present and stochastic restrictions are available. He showed that it outperformed the ordinary least squares, mixed estimator, ridge estimator, and other estimators considered in his study in terms of mean squared error sense. Ridge regression theory and an important shrinkage and model selection techniques with application to machine learning has been studied extensively for different models and settings by Saleh, A. K. *et al* [14, 15]. For more details about the shrinkage estimators, the reader is referred to S.E. Ahmed [16], Nkurunziza, S. *et. al* [17], Peng, L. *et.al* [18], and Saleh, A. K. [19], among others.

One common problem that researchers faced while fitting a multiple regression model using the ordinary least squares(OLS) method is the mulicolinearity, which occurs when some of the explanatory variables are correlated. This problem may cause insignificant regression coefficients or some of the coefficients have unexpected signs. There are many estimation methods proposed to improve the OLS estimators. For instance, Hoerl and Kennard [20] proposed the ridge estimate for the OLS estimator. Liu K. [21] introduced a biased estimate in linear regression. A modified version of Liu estimator was proposed by Li and Yang [22]. Yüzbaşı, B. *et.al* [23] proposed the pretest and shrinkage-type ridge regression estimators in case of linear models. Recently Yüzbaşı, B. *et.al* [24] proposed the pretest, shrinkage, and pretest-shrinkage Liu-type estimation in linear models. Babar, Iqra *et.al* [25] proposed new estimators for the shrinkage parameter of Liu estimator based on quantile of the regression coefficients. They showed the new estimator outperformed the existing estimators in terms of mean squared error and absolute error. Arashi M. *et al* [26] proposed an improved Liu-type unrestricted, restricted, pretest, shrinkage, and positive shrinkage estimators for the regression parameter vector of coefficients. They showed the superiority of the proposed method analytically and numerically. With respect to robust regression, Arashi, M. *et al* [27] defined the Liu-type rank-based estimators. They examined the asymptotic behavior of the estimators, and provided the proposed estimators’ superiority requirements for the biasing parameters, and supported their findings by numerical calculations. Arashi, M. *et al* [28] proposed the ridge estimator for high-dimensional multicollinear data. They proved the consistency and derived some asymptotic properties of the proposed estimators and applied it to simulation experiments and real data set. Arashi, M. *et al* [29] proposed a re-scaled LASSO for multicollinear situations. Their numerical analysis has demonstrated that the scaled LASSO performs frequently better than the LASSO and elastic net while being comparable to other sparse modeling techniques. Arashi, M *et al* [30] developed an improved ridge approach for the genome regression modeling and used a rank ridge estimator for parameter estimation and prediction when multicollinearity presents with outliers in the data set.

In this manuscript, we aim to propose efficient estimators for the large-scale effect parameter vector (***β***) in the CA model when it is suspected that some of the coefficients are not significant. So, we partition the (*p* × 1) parameter vector ***β*** as (***β***_1_, ***β***_2_), where ***β***_1_ is a (*p*_1_ × 1) vector, which is considered as the coefficients of the main effect, ***β***_2_ is a (*p*_2_ × 1) vector as the unimportant or nuisance parameters, and *p*_1_ + *p*_2_ = *p*. We are primarily interested in estimating ***β***_1_ when ***β***_2_ is suspected to be zero or close to zero. In some cases, the full model estimator may be highly variable and difficult to interpret, and the submodel estimator may result in a large biased and under-fitted estimator. To overcome this issue, we considered the Liu-type pretest, shrinkage and positive shrinkage estimators.

The rest of the paper is organized as follows in accordance with our goals. Section 2 provides a brief overview of the CA model. The maximum likelihood estimator of the CA model parameters are given in Section 3. In Section 4, we proposed the Liu-type estimators, and discussed the asymptotic properties in terms of bias, quadratic bias, and quadratic risks in Section 5. We compared the array of estimators using Monte Carlo simulation and real data example in Section 6. Some conclusions are given in Section 7.

## 2 Conditional autoregressive model

Assume, in accordance with Cressie and Wikle [31], that there are (*n*) spatial cites (usually referred as locations, geographical areas, etc). The collection of theses cites is known as a lattice indicated by the notation ***S*** = {*s*_1_, *s*_2_, …, *s*_*n*_}. For the *i*^*th*^ cite *s*_*i*_, a set of neighboring cites, denoted by *N*(*s*_*i*_) is defined as *N*(*s*_*i*_) = {*s*_*j*_ : *j* is a neighbor of *i*}, *j* = 1, 2, …, *n* in which a neighborhood structurer is defined based on a certain metric. For example, two sites are rook-based neighbors in a regular lattices if they have common boundaries, while it is a queen-based neighbors if the two sites have common boundaries and corners. Let ***Y***_*n*_(***s***) = {*Y*(*s*_1_), *Y*(*s*_2_), …, *Y*(*s*_*n*_))} be a vector of observations that collected at sites {*s*_1_, *s*_2_, …, *s*_*n*_}, and ***X***(*s*_*i*_) = ***X***_*i*_ = (*X*_1*i*_, *X*_2*i*_, …, *X*_*pi*_)′ be the set of covarites, and ***β*** = (*β*_1_, *β*_2_, …, *β*_*p*_)′ is a *p* × 1 vector of parameters, known as the large-scale effect on ***Y***_*n*_(***s***).

We will assume that ***Y***_*n*_(***s***) is continuous, and follows a Gaussian process with mean ***μ***(***s***) = *E*(***Y***_*n*_(***s***)) = ***X***′(***s***)***β*** and covariance matrix *Var*(***Y***) = *σ*^2^(***I***_*n*_ − ρW*)^−1^ ***D***, where *σ*^2^ > 0, *ρ* is the spatial dependence parameter, $W^{*}=\{\frac{w_{ij}}{w_{i+}}\}$ with *w*_*ij*_ = 1 if sites *i*, *j* (*i* ≠ *j*) are neighbors to each other, *w*_*ij*_ = 0 otherwise, *w*_*ii*_ = 0, $w_{i+}=\sum_{i=1}^{n}w_{ij}$, W* is called the standardized proximity matrix, and ***D*** is a diagonal matrix with $d_{ii}=\frac{1}{w_{i+}}$. For simplicity, the covariate vectors for all sites will be consolidated into a design matrix ***X***(***s***), and all subscripts (*n*, *s*) will be removed unless we need to present them explicitly. That is, the data on the lattice ***s*** will be denoted by (***Y***, ***X***). Following Besag *et al* [32], the Conditional Autoregressive (CA) model follows a multivariate Gaussian (Normal) distribution as ***Y*** ∼ *N*_*n*_(***Xβ***, ***V***_*n*_), where ***V***_*n*_ = *σ*^2^(***I***_*n*_−ρW*)^−1^ ***D***. In regression context, the CA model is given by:


$$\begin{array}{ccc} Y & = & X\beta +\epsilon , \end{array}$$ (1)

where ***ϵ*** ∼ *N*_*n*_(**0**, *σ*^2^***V***_*n*_). The model is known as a conditional autoregressive regression model because the mean and variance of *Y*(*s*_*i*_) can be written in a conational form, as follows:


$$E\left\{Y\left(s_{i}\right)|Y\left(s_{j}\right),i\neq j\right\}=X^{\prime }\left(s_{i}\right)\beta +\rho \sum_{i=1}^{n}w_{ij}(Y\left(s_{j}\right)-X^{\prime }\left(s_{j}\right)\beta ),$$

$$Var\left(Y\left(s_{i}\right)|Y\left(s_{j}\right),i\neq j\right)=\sigma_{i}^{2}=\frac{\sigma^{2}}{w_{i+}}.$$

## 3 The maximum likelihood estimation

The maximum likelihood estimators (MLEs) of the parameter vector ***β***, *σ*^2^, and the spatial dependence parameter *ρ* are derived by a two-step profile-likelihood procedure, see Cressie [33]. We fix the parameter *ρ* at first, then solve the log-likelihood equation, and plug $\hat{\beta }(\rho )$, $\hat{\sigma^{2}}(\rho )$ back in the log-likelihood to find the MLE of *ρ*, which is denoted by $\hat{\rho }$. The MLEs of ***β*** and *σ*^2^ are given by:

$$\begin{array}{ccc} \hat{\beta }\left(\rho \right) & = & {\left(X^{\prime }{\hat{V_{n}}}^{-1}X\right)}^{-1}X^{\prime }{\hat{V_{n}}}^{-1}Y, \end{array}$$ (2)

$$\begin{array}{ccc} \hat{\sigma^{2}}\left(\rho \right) & = & {\left(Y-X\hat{\beta }\left(\rho \right)\right)}^{\prime }{\hat{V_{n}}}^{-1}(Y-X\hat{\beta }\left(\rho \right)). \end{array}$$ (3)

Then, the MLE of *ρ* is a solution of the log-likelihood function that maximizes L*(*ρ*) see Ord [34], where

$$\begin{array}{ccc} L^{*}\left(\rho \right) & = & -\frac{n}{2}log[\frac{{\left(Y-X\hat{\beta }\left(\rho \right)\right)}^{\prime }{\hat{V_{n}}}^{-1}\left(Y-X\hat{\beta }\left(\rho \right)\right)}{n}]-\frac{1}{2}log(|\hat{V_{n}|}) \end{array}$$ (4)

Finally, we obtain the MLEs of ***β*** and *σ*^2^. We denote to the MLEs of (***β***, *σ*^2^, *ρ*) by $\hat{\vartheta }=(\hat{\beta },\hat{\sigma^{2}},\hat{\rho })$. Mardia and Marshall [35] proved the consistency and asymptotic normality of $\hat{\vartheta }$ which leads to the asymptotic normality of the large-scale parameter vector $\hat{\beta }$.

## 4 Efficient estimation strategies

Consider the following multiple linear regression model

$$\begin{array}{ccc} Y & = & X\beta +\epsilon , \end{array}$$ (5)

where *ϵ* ∼ *N*_*n*_(0, *σ*^2^***I***_*n*_). The ordinary least square estimators of ***β***, denoted by ${\hat{\beta }}^{OLS}$ is given by (***X***′***X***)^−1^***X***′***Y*** enjoys some good properties. However, when multicollinearity exits, the entries of (***X***′***X***)^−1^ become large, which cause a large variation of ${\hat{\beta }}^{OLS}$. To overcome the problem of multicollinearity, Hoerl and Kennard [20] proposed the ridge estimator which is given by:

$$\begin{array}{ccc} {\hat{\beta }}^{Ridge} & = & {\left(X^{\prime }X+kI_{p}\right)}^{-1}X^{\prime }Y, \end{array}$$ (6)

where *k* > 0, Note that if *k* = 0, ${\hat{\beta }}^{Ridge}={\hat{\beta }}^{OLS}$, and if *k* → ∞, ${\hat{\beta }}^{Ridge}=0$. Later on, Liu [21] proposed a biased estimator to deal with multicollinearity, which benefits form both the ridge estimator and shrinkage estimator, it is denoted by ${\hat{\beta }}^{LU}$, and given by:

$$\begin{array}{ccc} {\hat{\beta }}^{LU} & = & {\left(X^{\prime }X+I_{p}\right)}^{-1}(X^{\prime }Y+d{\hat{\beta }}^{OLS}) \end{array}$$ (7)

$$\begin{array}{cc} = & {\left(X^{\prime }X+I_{p}\right)}^{-1}(X^{\prime }X+dI_{p}){\hat{\beta }}^{OLS}, \end{array}$$ (8)

where 0 < *d* < 1, known as the biasing parameter. Obviously, when *d* = 1, ${\hat{\beta }}^{LU}={\hat{\beta }}^{OLS}$. In the next subsection we introduce the Liu estimator for for the CA model.

### 4.1 Liu estimators for the CA model

Generally speaking, subjective information about the importance of a certain regression coefficients is available. Such information divides the *p* × 1 regression parameter vector as ***β*** = (***β***_1_, ***β***_2_), where ***β***_1_, ***β***_2_ are of dimensions *p*_1_ × 1 and *p*_2_ × 1, respectively, with *p* = *p*_1_ + *p*_2_. Also, the *n* × *p* design matrix is partitioned as ***X*** = (***X***_1_, ***X***_2_), where ***X***_1_ is an *n* × *p*_1_ and ***X***_2_ an *n* × *p*_2_ matrices. So, the model in (1) can be rewritten as:

$$\begin{array}{ccc} Y & = & X_{1}\beta_{1}+X_{2}\beta_{2}+\epsilon \end{array}$$ (9)

We are initially interested in estimating ***β***_1_ by removing ***β***_2_ when ***X***_2_ is insignificant to explain the variation in the response variable. Such information can be obtained either from some variable selection approaches or some uncertain prior information. In other words, we may consider testing a restriction given by:

$$\begin{array}{ccc} H_{0}:\beta_{2} & = & 0 \end{array}$$ (10)

Assuming we obtained information about ***X***_2_, then the candidate sub-model is given by:

$$\begin{array}{ccc} Y & = & X_{1}\beta_{1}+\epsilon . \end{array}$$ (11)

The MLE of ***β***_1_ for the previous model in (11) can be easily obtained in a similar manners as we got $\hat{\beta }$ in (2), and is given by:

$$\begin{array}{ccc} {\hat{\beta_{1}}}^{SM} & = & {\left(X_{1}^{\prime }{\hat{V_{n}}}^{-1}X_{1}\right)}^{-1}X_{1}^{\prime }{\hat{V_{n}}}^{-1}Y. \end{array}$$ (12)

For the model in (9), the MLE of ***β***_1_ can be obtained by maximizing the log-likelihood given by

$$\begin{array}{ccc} l^{*}\left(\beta_{1},\beta_{2}\right) & = & -\frac{1}{2\sigma^{2}}{\left(Y-X_{1}\beta_{1}-X_{2}\beta_{2}\right)}^{\prime }V_{n}^{-1}(Y-X_{1}\beta_{1}-X_{2}\beta_{2}) \end{array}$$

By setting $\frac{\partial l^{*}(\beta_{1},\beta_{2})}{\partial \beta_{1}}=0$ and $\frac{\partial l^{*}(\beta_{1},\beta_{2})}{\partial \beta_{2}}=0$, then solve the two equations to get:

$$\begin{array}{ccc} \hat{\beta_{1}} & = & {\left(X_{1}^{\prime }A_{2}X_{1}\right)}^{-1}X_{1}^{\prime }A_{2}Y \end{array}$$ (13)

where $A_{2}={\hat{V_{n}}}^{-1}-{\hat{V_{n}}}^{-1}X_{2}(X_{2}^{\prime }{\hat{V_{n}}}^{-1}X_{2}{)}^{-1}X_{2}^{\prime }{\hat{V_{n}}}^{-1}$, and $\hat{\beta_{2}}$ has the same formula by interchanging the indices 1 and 2. Note that, $\hat{\beta_{1}}$ can be also written in terms of ${\hat{\beta_{1}}}^{SM}$ as follows:

$$\begin{array}{ccc} \hat{\beta_{1}} & = & {\hat{\beta_{1}}}^{SE}-{\left(X_{1}^{\prime }{\hat{V_{n}}}^{-1}X_{1}\right)}^{-1}(X_{1}^{\prime }{\hat{V_{n}}}^{-1}X_{2})\hat{\beta_{2}} \end{array}$$ (14)

We define the Liu estimator of ***β***_1_ as follows:

$$\begin{array}{ccc} {\hat{\beta_{1}}}^{LU} & = & {\left(X_{1}^{\prime }A_{2}X_{1}+I_{p_{1}}\right)}^{-1}(X_{1}^{\prime }A_{2}X_{1}+dI_{p_{1}})\hat{\beta_{1}}, \end{array}$$ (15)

where 0 < *d* < 1. We will refer to the estimator in ${\hat{\beta_{1}}}^{LU}$ in (15) as the full model estimator of ***β***_1_. The Liu estimator of the sub-model in (11) is defined as follows:

$$\begin{array}{ccc} {\hat{\beta_{1}}}^{LUS} & = & {\left(X_{1}^{\prime }{\hat{V_{n}}}^{-1}X_{1}+I_{p_{1}}\right)}^{-1}(X_{1}^{\prime }{\hat{V_{n}}}^{-1}X_{1}+d_{s}I_{p_{1}}){\hat{\beta_{1}}}^{SM}, \end{array}$$ (16)

where 0 < *d*_*s*_ < 1. In fact, under the null hypothesis in (10), ${\hat{\beta_{1}}}^{LUS}$ performs better than ${\hat{\beta_{1}}}^{LU}$ or when ***β***_2_ closes to **0**, but when ***β***_2_ starts moving away from the null space, ${\hat{\beta_{1}}}^{LUS}$ becomes inefficient, while ${\hat{\beta_{1}}}^{LU}$ remains consistent.

### 4.2 The pretest and shrinkage Liu-type estimators

The pretest Liu-type estimator of ***β***_1_ depends on testing the null hypothesis in (10). It chooses ${\hat{\beta_{1}}}^{LU}$ if the hypothesis is rejected at *α*−level of significance, and ${\hat{\beta_{1}}}^{LUS}$ otherwise. It is denoted by ${\hat{\beta_{1}}}^{PTL}$ and given by:

$$\begin{array}{ccc} {\hat{\beta_{1}}}^{PTL} & = & {\hat{\beta_{1}}}^{LU}-({\hat{\beta_{1}}}^{LU}-{\hat{\beta_{1}}}^{LUS})I\left(L_{n}\leq l_{n,\alpha }\right), \end{array}$$ (17)

where *I*(.) is the indicator function, *L*_*n*_ is a suitable test statistics for testing *H*_0_ in (10), and is given by: $L_{n}=\frac{\left[{\hat{\beta_{2}}}^{\prime }(X_{2}^{\prime }A_{1}X_{2})\hat{\beta_{2}}\right]}{S^{2}}$, $S^{2}=\frac{{\left(Y-X{\hat{\beta }}^{LU}\right)}^{\prime }{\hat{V_{n}}}^{-1}\left(Y-X{\hat{\beta }}^{LU}\right)}{n-p}$, *l*_*n*, *α*_ is the *α*−critical value of the distribution of the tests statistics *L*_*n*_, ***A***_1_ is defined in a similar way as ***A***_2_, and *S*^2^ is an estimator of *σ*^2^. The test statistics *L*_*n*_ follows a chi-square distribution with (*p*_2_) degrees of freedom under the null hypothesis. ${\hat{\beta_{1}}}^{PTL}$ is a binary choice between ${\hat{\beta_{1}}}^{LU}$ and ${\hat{\beta_{1}}}^{LUS}$, it chooses ${\hat{\beta_{1}}}^{LU}$ if *H*_0_ is rejected and ${\hat{\beta_{1}}}^{LUS}$ if not. The Liu-type shrinkage estimator provides a smoother weighting than ${\hat{\beta_{1}}}^{PTL}$. It is denoted by ${\hat{\beta_{1}}}^{SL}$, and given by:

$$\begin{array}{ccc} {\hat{\beta_{1}}}^{SL} & = & {\hat{\beta_{1}}}^{LUS}+\left({\hat{\beta_{1}}}^{LU}-{\hat{\beta_{1}}}^{LUS}\right)\left(1-\left(p_{2}-2\right)L_{n}^{-1}\right),\;p_{2}\geq 2. \end{array}$$ (18)

However, ${\hat{\beta_{1}}}^{SL}$ may experience an over-shrinkage problem, and produce unexpected signs of some of coefficients when *p*_2_ − 2 > *L*_*n*_. This issue was handled by the Liu-type positive shrinkage estimation of ***β***_1_, which is defined as:

$$\begin{array}{ccc} {\hat{\beta_{1}}}^{PSL} & = & {\hat{\beta_{1}}}^{LUS}+\left({\hat{\beta_{1}}}^{LU}-{\hat{\beta_{1}}}^{LUS}\right){\left(1-\left(p_{2}-2\right)L_{n}^{-1}\right)}^{+}, \end{array}$$ (19)

where *u*^+^ = *max*(0, *u*).

## 5 Asymptotic results

In this section, we study the asymptotic behaviour of the proposed estimators assuming a sequence of local alternatives {*H*_(*n*)_} given by:

$$\begin{array}{c} H_{\left(n\right)}:\beta_{2(n)}=\beta_{2}=n^{-\frac{1}{2}}\xi , \end{array}$$ (20)

where ***ξ*** is a *p*_2_ × 1 fixed and known vector. Clearly, if ***ξ*** = **0**, the local alternatives in (20) reduces to (10).

Let $\beta_{1}^{*}$ be any of the proposed estimators of ***β***_1_, and ***M*** be a *p*_1_ × *p*_1_ positive definite weight matrix. Define the cumulative distribution function of ${\hat{\theta }}_{n}^{*}=\sqrt{n}(\beta_{1}^{*}-\beta_{1})$ by $F(x)={\text{lim}}_{n\to \infty }P_{H_{\left(n\right)}}({\hat{\theta }}^{*}\leq x)$, and the quadratic loss function of $\beta_{1}^{*}$ as

$$\begin{array}{ccc} L(\beta_{1}^{*},\beta_{1}) & = & n{\left(\beta_{1}^{*}-\beta_{1}\right)}^{\prime }M\left(\beta_{1}^{*}-\beta_{1}\right)\\ & = & tr\{M(n\left(\beta_{1}^{*}-\beta_{1}\right){\left(\beta_{1}^{*}-\beta_{1}\right)}^{\prime })\}, \end{array}$$

where *tr*(***A***) is the trace of the matrix ***A***. If ${\hat{\theta }}_{n}^{*}\;\underset{\to }{D}\;{\hat{\theta }}^{*}$, where $\underset{\to }{D}$ denotes to the convergence in distribution, then the asymptotic quadratic risk (AR) of $\beta_{1}^{*}$ is defined as:

$$\begin{array}{ccc} AR(\beta_{1}^{*},M) & = & E\{{\hat{\theta }}^{*\prime }M{\hat{\theta }}^{*}\}\\ & = & \int \left(x^{\prime }Mx\right)dF\left(x\right) \end{array}$$ (21)

The asymptotic joint normality of the sub and full models Liu estimators is the main tool in deriving the AR expressions, we list two theorems below to find these expressions. Assuming the assumptions of theorem 2 of Mardia and Marshall [35] and the following:

-
${\underset{X_{n}}{\text{max}}}_{1\leq i\leq n}\;x_{i}^{\prime }(X_{n}^{\prime }{\hat{V_{n}}}^{-1}X_{n}{)}^{-1}x_{i}\to 0$ as *n* → ∞, where ***x***_*i*_ is the *i*^*th*^ row of ***X***_*n*_
-
$\underset{n\to \infty }{\text{lim}}C_{n}=\underset{n\to \infty }{\text{lim}}\left(\frac{X_{n}^{\prime }{\hat{V_{n}}}_{n}^{-1}}{n}\right)=C$ , where ***C*** is a finite positive definite matrix.
-
$\underset{n\to \infty }{\text{lim}}\;G_{n}\left(d\right)=G_{d}$ , where ***G***_*n*_(*d*) = (***C***_*n*_ + ***I***_*p*_)^−1^(***C***_*n*_ + *d**I***_*p*_), and ***G***_*d*_ = (***C*** − ***I***_*p*_)^−1^(***C*** + *d**I***_*p*_)

**Theorem 1** *If* 0 < *d* < 1, *and* |***C***| ≠ 0, *then* $\sqrt{n}\left({\hat{\beta }}^{LU}-\beta \right)\overset{d}{\to }N_{p}\left(-(1-d)(C+I_{p}{)}^{-1}\beta ,\sigma^{2}B\right)$, *where* $B=G_{d}C^{-1}G_{d}^{\prime }$, *and* $\underset{\to }{d}$ *denotes to the convergence in distribution*.

**Proof**: Note that $I_{p}={\left(X^{\prime }{\hat{V_{n}}}^{-1}X+I_{p}\right)}^{-1}\left(X^{\prime }{\hat{V_{n}}}^{-1}X+I_{p}\right)$, so ${\left(X^{\prime }{\hat{V_{n}}}^{-1}X+I_{p}\right)}^{-1}(X^{\prime }{\hat{V_{n}}}^{-1}X)=I_{p}-{\left(X^{\prime }{\hat{V_{n}}}^{-1}X+I_{p}\right)}^{-1}$

$$\begin{array}{ccc} \sqrt{n}({\hat{\beta }}^{LU}-\beta ) & = & \sqrt{n}\{{\left(X^{\prime }{\hat{V_{n}}}^{-1}X+I_{p}\right)}^{-1}(X^{\prime }{\hat{V_{n}}}^{-1}X+dI_{p})\hat{\beta }-\beta \}\\ & = & \sqrt{n}\{[{\left(X^{\prime }{\hat{V_{n}}}^{-1}X+I_{p}\right)}^{-1}\left(X^{\prime }{\hat{V_{n}}}^{-1}X\right)+d{\left(X^{\prime }{\hat{V_{n}}}^{-1}X+I_{p}\right)}^{-1}\\ & & ]\hat{\beta }-\beta \}\\ & = & \sqrt{n}\{[I_{p}-{\left(X^{\prime }{\hat{V_{n}}}^{-1}X+I_{p}\right)}^{-1}+d{\left(X^{\prime }{\hat{V_{n}}}^{-1}X+I_{p}\right)}^{-1}]\hat{\beta }-\beta \}\\ & = & \sqrt{n}\{(\hat{\beta }-\beta )-\left(1-d\right){\left(X^{\prime }{\hat{V_{n}}}^{-1}X+I_{p}\right)}^{-1}\hat{\beta }\} \end{array}$$

which is a linear combination of $\hat{\beta }$. Hence, by Mardia and Marshall theorem [35], and as *n* → ∞, $\sqrt{n}\left({\hat{\beta }}^{LU}-\beta \right)$ converges in distribution to multivariate Gaussian distribution with: *Mean* = −(1 − *d*)(***C***−***I***_*p*_)^−1^***β***, and

$$\begin{array}{ccc} Var({\hat{\beta }}^{LU}) & = & {\left(C+I_{p}\right)}^{-1}\left(C+dI_{p}\right)Var\left(\hat{\beta }\right)\left(C+dI_{p}\right){\left(C+I_{p}\right)}^{-1}\\ & = & \sigma^{2}{\left(C+I_{p}\right)}^{-1}\left(C+dI_{p}\right)C^{-1}\left(C+dI_{p}\right){\left(C+I_{p}\right)}^{-1}\\ & = & \sigma^{2}G_{d}C^{-1}G_{d}^{\prime } \end{array}$$

**Theorem 2** *Let* ${\hat{\theta }}_{n}^{\left(1\right)}=\sqrt{n}\left({\hat{\beta_{1}}}^{LU}-\beta_{1}\right)$, ${\hat{\theta }}_{n}^{\left(2\right)}=\sqrt{n}\left({\hat{\beta_{1}}}^{LUS}-\beta_{1}\right)$, ${\hat{\theta }}_{n}^{\left(3\right)}=\sqrt{n}\left({\hat{\beta_{1}}}^{LU}-{\hat{\beta_{1}}}^{LUS}\right)$. *Under the previous assumptions, the sequence of local alternatives in* (20), *and as* *n* → ∞, *we have*:

1.
$\left(\begin{array}{c} {\hat{\theta }}_{n}^{\left(1\right)}\\ {\hat{\theta }}_{n}^{\left(3\right)} \end{array}\right)\overset{d}{\to }N\left(\left(\begin{array}{c} -\lambda_{11.2}\\ \pi \end{array}\right),\sigma^{2}\left(\begin{array}{cc} B_{11.2}^{-1} & B^{*}\\ B^{*} & B^{*} \end{array}\right)\right)$

2.
$\left(\begin{array}{c} {\hat{\theta }}_{n}^{\left(3\right)}\\ {\hat{\theta }}_{n}^{\left(2\right)} \end{array}\right)\overset{d}{\to }N\left(\left(\begin{array}{c} \pi \\ -\gamma \end{array}\right),\sigma^{2}\left(\begin{array}{cc} B^{*} & 0\\ 0 & B_{11}^{-1} \end{array}\right)\right)$ ,

where $B=\left(\begin{array}{cc} B_{11} & B_{12}\\ B_{21} & B_{22} \end{array}\right)$, $C=\left(\begin{array}{cc} C_{11} & C_{12}\\ C_{21} & C_{22} \end{array}\right)$,

***γ*** = − (**λ**_11.2_ − ***π***), $\pi =(C_{11}+I_{p_{1}}{)}^{-1}(C_{11}+dI_{p_{1}})\xi$, $B^{*}=G_{d}^{11}C_{12}B_{22.1}^{-1}C_{21}G_{d}^{11}$, $B_{22.1}=B_{22}-B_{21}B_{11}^{-1}B_{12}$, $G_{d}^{11}=(C_{11}+I_{p_{1}}{)}^{-1}(C_{11}+dI_{p_{1}})$, $\lambda =-(1-d)(C+I_{p}{)}^{-1}\beta =\left(\begin{array}{c} \lambda_{1}\\ \lambda_{2} \end{array}\right)$, and $\lambda_{11.2}=\lambda_{1}-C_{12}C_{22}^{-1}\left((\beta_{2}-\xi )-\lambda_{2}\right)$ is the conditional distribution mean of ***β***_1_ given $\beta_{2}=0_{p_{2}}$.

The proof of Theorem (2) is similar to the proof of Theorem (1) with little modification. Also, we refer to Bahadır Y. *et al* [36] for a similar proof.

### 5.1 Asymptotic distributional and quadratic bias of the estimators

The asymptotic distributional bias expressions, denoted by $AB(\beta_{1}^{*})$, where $\beta_{1}^{*}$ is any of the the prosed estimators, are given in the following theorem.

**Theorem 3** *The AB expressions are*:

1.
$AB({\hat{\beta_{1}}}^{LU})=-\lambda_{11.2}$ ,
2.
$AB({\hat{\beta_{1}}}^{LUS})=-\gamma$ ,
3.
$AB({\hat{\beta_{1}}}^{PTL})=-\lambda_{11.2}-\pi H_{p_{2}+2}(\chi_{p_{2}(\alpha )}^{2};\Delta )$ ,
4.
$AB({\hat{\beta_{1}}}^{SL})=-\lambda_{11.2}-(p_{2}-2)\pi E(\chi_{p_{2}+2}^{-2}(\Delta ))$ ,
5.
$AB({\hat{\beta_{1}}}^{PSL})=-\lambda_{11.2}-\pi H_{p_{2}+2}(\chi_{p_{2}(\alpha )}^{2};\Delta )-(p_{2}-2)\pi E\{\chi_{p_{2}+2}^{-2}(\Delta )I\left(\chi_{p_{2}+2}^{2}(\Delta )>p_{2}-2\right)\}$ ,

where $\Delta =\frac{\xi^{\prime }B_{22.1}^{-1}\xi }{\sigma^{2}}$, *H*_*n*_(*x*;*Δ*) is the cumulative distribution function of non-cental chi-square distribution with (*n*) degrees of freedom and a non-centrality parameter Δ, and $E\left(\chi_{n}^{-2i}(\Delta )\right)=\int_{0}^{\infty }x^{-2i}dH_{n}(x;\Delta )$. To proof the previous theorem we use the following theorem. The proof can be found in [37].

**Theorem 4** *Let* ***y*** = (*y*_1_, *y*_2_, …, *y*_*q*_)′ be *N*_*q*_(***μ***, **Σ**), *and let* *ϕ* *be any measurable function, then* $E\left(y\phi (yy^{\prime })\right)=\mu E\left(\chi_{q+2}^{2}(\Delta )\right)$, where $\chi_{n}^{2}(\Delta )$ is the chi-square random variable with (*n*) degrees of freedom and $\Delta =\frac{\mu^{\prime }\mu }{2}$ is the non-centrality parameter.

**Proof of Theorem (3):**

1.
$AB({\hat{\beta_{1}}}^{LU})=-\lambda_{11.2}$ by Theorem (2)-part (2).
2. Note that: ${\hat{\beta_{1}}}^{LUS}={\hat{\beta_{1}}}^{LU}+{\left(X_{1}^{\prime }{\hat{V_{n}}}^{-1}X_{1}+I_{p_{1}}\right)}^{-1}\left(X_{1}^{\prime }{\hat{V_{n}}}^{-1}X_{1}+dI_{p_{1}}\right)\left(X_{1}^{\prime }{\hat{V_{n}}}^{-1}X_{2}\right)\hat{\beta_{2}}$.
  Therefore,
  $$\begin{array}{ccc} AB({\hat{\beta_{1}}}^{LUS}) & = & E\{\underset{n\to \infty }{\text{lim}}\sqrt{n}({\hat{\beta_{1}}}^{LU}-\beta_{1})\}\\ & + & E\{\underset{n\to \infty }{\text{lim}}{\left(X_{1}^{\prime }{\hat{V_{n}}}^{-1}X_{1}+I_{p_{1}}\right)}^{-1}(X_{1}^{\prime }{\hat{V_{n}}}^{-1}X_{1}+dI_{p_{1}})\\ & & \left(X_{1}^{\prime }{\hat{V_{n}}}^{-1}X_{2})\sqrt{n}\hat{\beta_{2}}\right\}\\ & = & -\lambda_{11.2}+G_{d}^{11}C_{12}\xi \\ & = & -(\lambda_{11.2}-\pi )=-\gamma . \end{array}$$
3.
$$\begin{array}{ccc} AB({\hat{\beta_{1}}}^{PTL}) & = & E\{\underset{n\to \infty }{\text{lim}}\sqrt{n}({\hat{\beta_{1}}}^{PTL}-\beta_{1})\}\\ & = & E\{\underset{n\to \infty }{\text{lim}}\sqrt{n}({\hat{\beta_{1}}}^{LU}-({\hat{\beta_{1}}}^{LU}-{\hat{\beta_{1}}}^{LUS})I(L_{n}\leq l_{n,\alpha }))-\beta_{1}\}\\ & = & E\{\underset{n\to \infty }{\text{lim}}\sqrt{n}({\hat{\beta_{1}}}^{LU}-\beta_{1})\}\\ & - & E\{\underset{n\to \infty }{\text{lim}}\sqrt{n}({\hat{\beta_{1}}}^{LU}-{\hat{\beta_{1}}}^{LUS})I(L_{n}\leq l_{n,\alpha })\},\text{by}\;\text{Theorem}\left(4\right)\\ & = & -\lambda_{11.2}-\pi H_{p_{2}+2}\left(\chi_{p_{2}\left(\alpha \right)}^{2};\Delta \right) \end{array}$$
4. Note that ${\hat{\beta_{1}}}^{SL}$ can be written as
  $$\begin{array}{ccc} {\hat{\beta_{1}}}^{SL} & = & {\hat{\beta_{1}}}^{LUS}+({\hat{\beta_{1}}}^{LU}-{\hat{\beta_{1}}}^{LUS})(1-\left(p_{2}-2\right)L_{n}^{-1})\\ & = & {\hat{\beta_{1}}}^{LU}-({\hat{\beta_{1}}}^{LU}-{\hat{\beta_{1}}}^{LUS})\left(p_{2}-2\right)L_{n}^{-1}. \end{array}$$
  Therefore,
  $$\begin{array}{ccc} AB({\hat{\beta_{1}}}^{SL}) & = & E\{\underset{n\to \infty }{\text{lim}}\sqrt{n}({\hat{\beta_{1}}}^{SL}-\beta_{1})\}\\ & = & E\{\underset{n\to \infty }{\text{lim}}\sqrt{n}({\hat{\beta_{1}}}^{LU}-\beta_{1})\}\\ & - & E\{\underset{n\to \infty }{\text{lim}}\sqrt{n}({\hat{\beta_{1}}}^{LU}-{\hat{\beta_{1}}}^{LUS})\left(p_{2}-2\right)L_{n}^{-1}\}. \end{array}$$
  Using Theorem (2) and Theorem (4), we get: $AB({\hat{\beta_{1}}}^{SL})=-\lambda_{11.2}-(p_{2}-2)\pi E\left(\chi_{p_{2}+2}^{-2}(\Delta )\right)$
5. Note that ${\hat{\beta_{1}}}^{PSL}$ can be rewritten as:
  $$\begin{array}{ccc} {\hat{\beta_{1}}}^{PSL} & = & {\hat{\beta_{1}}}^{LUS}+({\hat{\beta_{1}}}^{LU}-{\hat{\beta_{1}}}^{LUS})(1-\left(p_{2}-2\right)L_{n}^{-1})I(L_{n}\geq p_{2}-2)\\ & = & {\hat{\beta_{1}}}^{LUS}+({\hat{\beta_{1}}}^{LU}-{\hat{\beta_{1}}}^{LUS})(1-\left(p_{2}-2\right)L_{n}^{-1})(1-I(L_{n}\leq p_{2}-2))\\ & = & {\hat{\beta_{1}}}^{SL}-({\hat{\beta_{1}}}^{LU}-{\hat{\beta_{1}}}^{LUS})(1-\left(p_{2}-2\right)L_{n}^{-1})I(L_{n}\leq p_{2}-2). \end{array}$$
  Therefore,
  $$\begin{array}{ccc} AB({\hat{\beta_{1}}}^{PSL}) & = & E\{\underset{n\to \infty }{\text{lim}}\sqrt{n}({\hat{\beta_{1}}}^{SL}-\beta_{1})\}\\ & - & E\{\underset{n\to \infty }{\text{lim}}\sqrt{n}({\hat{\beta_{1}}}^{LU}-{\hat{\beta_{1}}}^{LUS})(1-\left(p_{2}-2\right)L_{n}^{-1})\\ & & I(L_{n}\leq p_{2}-2)\}\\ & = & AB\left({\hat{\beta_{1}}}^{SL}\right)-\left(p_{2}-2\right)\pi E(\chi_{p_{2}+2}^{-2}\left(\Delta \right))\\ & - & \pi E\{(1-\left(p_{2}-2\right)\chi_{p_{2}+2}^{-2}\left(\Delta \right))I\left(\chi_{p_{2}+2}^{2}\left(\Delta \right)<p_{2}-2\right)\}\\ & = & -\lambda_{11.2}-\left(p_{2}-2\right)\pi E(\chi_{p_{2}+2}^{-2}\left(\Delta \right))-\left(p_{2}-2\right)\pi E(\chi_{p_{2}+2}^{-2}\left(\Delta \right))\\ & + & \pi E\{\left(p_{2}-2\right)\chi_{p_{2}+2}^{-2}\left(\Delta \right)I(\chi_{p2+2}^{2}\left(\Delta \right)\leq p_{2}-2)\}\\ & = & -\lambda_{11.2}-\pi H_{p_{2}+2}\left(\chi_{p_{2}\left(\alpha \right)}^{2};\Delta \right)\\ & - & \pi \left(p_{2}-2\right)E\{\chi_{p_{2}+2}^{-2}\left(\Delta \right)I(\chi_{p2+2}^{2}\left(\Delta \right)>p_{2}-2)\}. \end{array}$$

The asymptotic bias expressions are in vector format which can’t be used directly to compare the set of estimators. However, the asymptotic quadratic bias, which is a real number, can be used as a measure of the risk. Following Bahadir [36], the asymptotic quadratic risk for any estimator, denoted by $AQ(\hat{\beta_{1}^{*}})$, where $\hat{\beta_{1}^{*}}$ is any of the previous estimators, is defined as:

$$\begin{array}{ccc} AQ(\hat{\beta_{1}^{*}}) & = & {\left(AB(\hat{\beta_{1}^{*}})\right)}^{\prime }B_{11.2}(AB(\hat{\beta_{1}^{*}})) \end{array}$$ (22)

Consequently, the *AQ* expressions of the proposed estimators are given below:

1.
$AQ({\hat{\beta_{1}}}^{LU})=\lambda_{11.2}^{\prime }B_{11.2}\lambda_{11.2}$ ,
2.
$AQ({\hat{\beta_{1}}}^{LUS})=\gamma^{\prime }B_{11.2}\gamma$ ,
3.
$$\begin{array}{ccc} AQ({\hat{\beta_{1}}}^{PTL}) & = & \lambda_{11.2}^{\prime }B_{11.2}\lambda_{11.2}+\lambda_{11.2}^{\prime }B_{11.2}\pi H_{p_{2}+2}\left(\chi_{p_{2}\left(\alpha \right)}^{2};\Delta \right)\\ & + & \pi^{\prime }B_{11.2}\lambda_{11.2}H_{p_{2}+2}\left(\chi_{p_{2}\left(\alpha \right)}^{2};\Delta \right)\\ & + & \pi^{\prime }B_{11.2}\pi H_{p_{2}+2}{\left(\chi_{p_{2}\left(\alpha \right)}^{2};\Delta \right)}^{2}, \end{array}$$
4.
$$\begin{array}{ccc} AQ({\hat{\beta_{1}}}^{SL}) & = & \lambda_{11.2}^{\prime }B_{11.2}\lambda_{11.2}+\left(p_{2}-2\right)\lambda_{11.2}^{\prime }B_{11.2}\pi E(\chi_{p_{2}+2}^{-2}\left(\Delta \right))\\ & + & \left(p_{2}-2\right)\pi^{\prime }B_{11.2}\lambda_{11.2}E(\chi_{p_{2}+2}^{-2}\left(\Delta \right))\\ & + & {\left(p_{2}-2\right)}^{2}\pi^{\prime }B_{11.2}\pi {\left(E(\chi_{p_{2}+2}^{-2}\left(\Delta \right))\right)}^{2}, \end{array}$$
5.
$$\begin{array}{ccc} AQ({\hat{\beta_{1}}}^{PSL}) & = & \lambda_{11.2}^{\prime }B_{11.2}\lambda_{11.2}\\ & + & \left(\lambda_{11.2}^{\prime }B_{11.2}\pi +\pi^{\prime }B_{11.2}\lambda_{11.2}\right)\\ & & \left(H_{p_{2}+2}\left(\chi_{p_{2}\left(\alpha \right)}^{2};\Delta \right)+\left(p_{2}-2\right)E[\chi_{p_{2}+2}^{-2}\left(\Delta \right)I\left(\chi_{p2+2}^{2}\left(\Delta \right)>p_{2}-2\right)]\right)\\ & + & \pi^{\prime }B_{11.2}\pi \{H_{p_{2}+2}{\left(\chi_{p_{2}\left(\alpha \right)}^{2};\Delta \right)}^{2}\\ & + & \left(p_{2}-2\right)H_{p_{2}+2}\left(\chi_{p_{2}\left(\alpha \right)}^{2};\Delta \right)E(\chi_{p_{2}+2}^{-2}\left(\Delta \right))I\left(\chi_{p_{2}+2}^{2}\left(\Delta \right)>p_{2}-2\right)\}\\ & & [2+\left(p_{2}-2\right)E(\chi_{p_{2}+2}^{-2}\left(\Delta \right)I\left(\chi_{p_{2}+2}^{2}\left(\Delta \right)\right)>p_{2}-2)]. \end{array}$$

### 5.2 Asymptotic quadratic risk

The asymptotic quadratic risk (QR) can be used as a measure of relative performance with respect to the classical MLE of the full model estimator. To obtain the expressions of the QRs of the proposed estimators, we define the quadratic loss function as:

$$\begin{array}{ccc} L(\hat{\beta_{1}^{*}},\hat{\beta_{1}}) & = & \{n{\left(\hat{\beta_{1}^{*}}-\hat{\beta_{1}}\right)}^{\prime }\left(\hat{\beta_{1}^{*}}-\hat{\beta_{1}}\right)\}, \end{array}$$ (23)

where $\hat{\beta_{1}^{*}}$ as any of the proposed estimators, and $\hat{\beta_{1}}$ is the MLE of the model in (11). Also, the asymptotic covariance matrix (AC) of $\hat{\beta_{1}^{*}}$ is defined as:

$$\begin{array}{ccc} AC(\hat{\beta_{1}^{*}}) & = & E\{\underset{n\to \infty }{\text{lim}}n\left(\hat{\beta_{1}^{*}}-\hat{\beta_{1}}\right){\left(\hat{\beta_{1}^{*}}-\hat{\beta_{1}}\right)}^{\prime }\}. \end{array}$$ (24)

Finally, for any *p*_1_ × *p*_1_ positive definite matrix ***M***, the $QR(\hat{\beta_{1}^{*}})$ is defined as:

$$\begin{array}{ccc} QR(\hat{\beta_{1}^{*}}) & = & E\{\underset{n\to \infty }{\text{lim}}n\left(\hat{\beta_{1}^{*}}-\hat{\beta_{1}}\right)M{\left(\hat{\beta_{1}^{*}}-\hat{\beta_{1}}\right)}^{\prime }\}\\ & = & \text{tr}(MAC(\hat{\beta_{1}^{*}})), \end{array}$$ (25)

where tr(***W***) is the trace of the matrix ***W***. To derive the QR expressions we use the following theorem

**Theorem 5** *Let* ***y*** = (*y*_1_, *y*_2_, …, *y*_*q*_)′ be *N*_*q*_(***μ***, **Σ**), *and let* *ϕ* *be any measurable function, then* $E\left(yy^{\prime }\phi (y^{\prime }y)\right)=\mu \mu^{\prime }E\left(\chi_{q+4}^{2}(\Delta )\right)+\Sigma E\left(\phi (\chi_{q+2}^{2},(\Delta ))\right)$,

The proof can be found in [37].

**Theorem 6** *Under the assumptions of Theorem (2), the QR expressions are as follows*:

1.
$QR({\hat{\beta_{1}}}^{LU})=\sigma^{2}\;\text{tr}(MB_{11.2}^{-1})+\lambda_{11.2}^{\prime }M\lambda_{11.2}$ ,
2.
$QR({\hat{\beta_{1}}}^{LUS})=\sigma^{2}\;\text{tr}(MB_{11}^{-1})+\gamma^{\prime }M\gamma$ ,
3.
$$\begin{array}{ccc} QR({\hat{\beta_{1}}}^{PTL}) & = & QR\left({\hat{\beta_{1}}}^{LU}\right)+2\lambda_{11.2}^{\prime }MH_{p_{2}+2}\left(\chi_{p_{2}\left(\alpha \right)}^{2};\Delta \right)\\ & - & \text{tr}(MB^{*})H_{p_{2}+2}\left(\chi_{p_{2}\left(\alpha \right)}^{2};\Delta \right)\\ & + & \pi^{\prime }M\pi \{2H_{p_{2}+2}\left(\chi_{p_{2}\left(\alpha \right)}^{2};\Delta \right)-H_{p_{2}+4}\left(\chi_{p_{2}\left(\alpha \right)}^{2};\Delta \right)\}, \end{array}$$
4.
$$\begin{array}{ccc} QR({\hat{\beta_{1}}}^{SL}) & = & QR\left({\hat{\beta_{1}}}^{LU}\right)+2\left(p_{2}-2\right)\lambda_{11.2}M\pi E(\chi_{p_{2}+2}^{-2}\left(\Delta \right))\\ & - & \left(p_{2}-2\right)\;\text{tr}(MB^{*})[E(\chi_{p_{2}+2}^{-2}\left(\Delta \right)-\left(p_{2}-2\right)E(\chi_{p_{2}+2}^{-4}\left(\Delta \right)))]\\ & + & \left(p_{2}-2\right)\pi^{\prime }M\pi [2E(\chi_{p_{2}+2}^{-2}\left(\Delta \right))-2E(\chi_{p_{2}+4}^{-2}\left(\Delta \right))\\ & + & \left(p_{2}-2\right)E(\chi_{p_{2}+4}^{-4}\left(\Delta \right))], \end{array}$$
5.
$$\begin{array}{ccc} QR({\hat{\beta_{1}}}^{PSL}) & = & QR({\hat{\beta_{1}}}^{SL})\\ & + & 2\lambda_{11.2}^{\prime }M\pi E(\{(1-\left(p_{2}-2\right)\chi_{p_{2}+2}^{-2}\left(\Delta \right))\}I\left(\chi_{p_{2}+2}^{2}\left(\Delta \right)\leq p_{2}-2\right))\\ & + & \left(MB^{*}\right)E\{(1-\left(p_{2}-2\right)\chi_{p_{2}+2}^{-2}\left(\Delta \right))I\left(\chi_{p_{2}+2}^{-2}\left(\Delta \right)\leq p_{2}-2\right)\}\\ & - & 2\pi^{\prime }M\pi E\{(1-\left(p_{2}-2\right)\chi_{p_{2}+4}^{-2}\left(\Delta \right))I\left(\chi_{p_{2}+4}^{-2}\left(\Delta \right)\leq p_{2}-2\right)\}\\ & + & 2\pi^{\prime }M\pi E\{(1-\left(p_{2}-2\right)\chi_{p_{2}+2}^{-2}\left(\Delta \right)I\left(\chi_{p_{2}+2}^{-2}\left(\Delta \right)\leq p_{2}-2\right))\}\\ & - & {\left(p_{2}-2\right)}^{2}\;\text{tr}(MB^{*})E\{\chi_{p_{2}+2}^{-4}\left(\Delta \right)I\left(\chi_{p_{2}-2}^{2}\left(\Delta \right)\leq p_{2}-2\right)\}\\ & - & {\left(p_{2}-2\right)}^{2}\pi^{\prime }M\pi E\{\chi_{p_{2}+4}^{-4}\left(\Delta \right)I\left(\chi_{p_{2}-2}^{2}\left(\Delta \right)\leq p_{2}-2\right)\}\\ & + & \text{tr}(MB^{*})H_{p_{2}+2}\left(\chi_{p_{2}\left(\alpha \right)}^{2};\Delta \right)+\pi^{\prime }M\pi H_{p_{2}+4}\left(\chi_{p_{2}\left(\alpha \right)}^{2};\Delta \right) \end{array}$$

**Proof:**

1.
$$\begin{array}{ccc} AC({\hat{\beta_{1}}}^{LU}) & = & E\{\underset{n\to \infty }{\text{lim}}n\left({\hat{\beta_{1}}}^{LU}-\beta_{1}\right){\left({\hat{\beta_{1}}}^{LU}-\beta_{1}\right)}^{\prime }\}\\ & = & E\{\underset{n\to \infty }{\text{lim}}n{\hat{\theta }}_{n}^{\left(1\right)}{\hat{\theta }}_{n}^{\prime (1)}\},\;\text{by}\;\text{Theorem}\left(1\right)\\ & = & \sigma^{2}B_{11.2}^{-1}+\left(-\lambda_{11.2}\right){\left(-\lambda_{11.2}\right)}^{\prime }\\ & = & \sigma^{2}B_{11.2}^{-1}+\lambda_{11.2}\lambda_{11.2}^{\prime }.\;\text{Hence}\\ QR({\hat{\beta_{1}}}^{LU},M) & = & \text{tr}(MAC\left({\hat{\beta_{1}}}^{LU}\right))\\ & = & \text{tr}(M(\sigma^{2}B_{11.2}^{-1}+\lambda_{11.2}\lambda_{11.2}^{\prime }))\\ & = & \sigma^{2}\text{tr}(MB_{11.2}^{-1})+\lambda_{11.2}^{\prime }M\lambda_{11.2}. \end{array}$$
2.
$$\begin{array}{ccc} AC({\hat{\beta_{1}}}^{LUS}) & = & E\{\underset{n\to \infty }{\text{lim}}n\left({\hat{\beta_{1}}}^{LUS}-\beta_{1}\right){\left({\hat{\beta_{1}}}^{LUS}-\beta_{1}\right)}^{\prime }\}\\ & = & \sigma^{2}B_{11}^{-1}+\gamma \gamma^{\prime },\;\text{by}\;\text{Theorem}\left(1\right).\;\text{So}\\ QR({\hat{\beta_{1}}}^{LUS},M) & = & \text{tr}(MAC({\hat{\beta_{1}}}^{LUS}))\\ & = & \sigma^{2}\text{tr}(MB_{11}^{-1})+\gamma^{\prime }M\gamma . \end{array}$$
3.
$$\begin{array}{ccc} AC({\hat{\beta_{1}}}^{PTL}) & = & E\{\underset{n\to \infty }{\text{lim}}n\left({\hat{\beta_{1}}}^{PTL}-\beta_{1}\right){\left({\hat{\beta_{1}}}^{PTL}-\beta_{1}\right)}^{\prime }\}\\ & = & E\{\underset{n\to \infty }{\text{lim}}n[({\hat{\beta_{1}}}^{LU}-\beta_{1})-({\hat{\beta_{1}}}^{LU}-{\hat{\beta_{1}}}^{LUS})I\left(L_{n}\leq l_{n,\alpha }\right)]\\ & & {\left[\left({\hat{\beta_{1}}}^{LU}-\beta_{1}\right)-({\hat{\beta_{1}}}^{LU}-{\hat{\beta_{1}}}^{LUS})I\left(L_{n}\leq l_{n,\alpha }\right)\right]}^{\prime }\}\\ & = & E\{\underset{n\to \infty }{\text{lim}}n[{\hat{\theta }}_{n}^{\left(1\right)}-{\hat{\theta }}_{n}^{\left(3\right)}I\left(L_{n}\leq l_{n,\alpha }\right)][{\hat{\theta }}_{n}^{\left(1\right)}-{\hat{\theta }}_{n}^{\left(3\right)}\\ & & I\left(L_{n}\leq l_{n,\alpha }\right){]}^{\prime }\}\\ & = & E\{\underset{n\to \infty }{\text{lim}}n{\hat{\theta }}_{n}^{\left(1\right)}{\hat{\theta }}_{n}^{\prime (1)}\}-2E\{\underset{n\to \infty }{\text{lim}}n{\hat{\theta }}_{n}^{\left(1\right)}{\hat{\theta }}_{n}^{\prime (3)}I\left(L_{n}\leq l_{n,\alpha }\right)\}\\ & + & E\{\underset{n\to \infty }{\text{lim}}n{\hat{\theta }}_{n}^{\left(3\right)}{\hat{\theta }}_{n}^{\prime (3)}I\left(L_{n}\leq l_{n,\alpha }\right)\}\\ & = & E_{1}-2E_{2}+E_{3}, \end{array}$$
  where
  $$\begin{array}{ccc} E_{1} & = & E\{\underset{n\to \infty }{\text{lim}}n{\hat{\theta }}_{n}^{\left(1\right)}{\hat{\theta }}_{n}^{\prime (1)}\},\;\text{by}\;\text{part}\left(1\right)\\ & = & \sigma^{2}B_{11.2}^{-1}+\lambda_{11.2}\lambda_{11.2}^{\prime }, \end{array}$$
  $$\begin{array}{ccc} E_{2} & = & E\{\underset{n\to \infty }{\text{lim}}n{\hat{\theta }}_{n}^{\left(1\right)}{\hat{\theta }}_{n}^{\prime (3)}I\left(L_{n}\leq l_{n,\alpha }\right)\}\\ & = & E\{E\{\underset{n\to \infty }{\text{lim}}n{\hat{\theta }}_{n}^{\left(1\right)}{\hat{\theta }}_{n}^{\prime (3)}I\left(L_{n}\leq l_{n,\alpha }\right)\}|{\hat{\theta }}_{n}^{\left(3\right)}\}. \end{array}$$

Since ${\hat{\theta }}_{n}^{\left(i\right)}\overset{d}{\to }\theta^{\left(i\right)}$, *i* = 1, 2, 3, the conditional mean of ***θ***^(1)^|***θ***^(3)^ is given by: − **λ**_11.2_ + B*(B*)^−1^(*θ*^(3)^−***π***) = −**λ**_11.2_ + (***θ***^(3)^ − ***π***). Therefore,

$$\begin{array}{ccc} E_{2} & = & E\{\theta^{\left(3\right)}(-\lambda_{11.2}+\left(\theta^{\prime (3)}-\pi^{\prime }\right))I\left(\chi_{p_{2}}^{2}\leq c_{\alpha }\right)\},\;\text{by}\;\text{Theorem}\left(5\right)\\ & = & -\lambda_{11.2}\pi H_{p_{2}+2}\left(\chi_{p_{2}\left(\alpha \right)}^{2};\Delta \right)+\pi \pi^{\prime }H_{p_{2}+4}\left(\chi_{p_{2}\left(\alpha \right)}^{2};\Delta \right)\\ & + & B^{*}H_{p_{2}+2}\left(\chi_{p_{2}\left(\alpha \right)}^{2};\Delta \right)-\lambda_{11.2}\pi \pi^{\prime }H_{p_{2}+2}\left(\chi_{p_{2}\left(\alpha \right)}^{2};\Delta \right) \end{array}$$

$$\begin{array}{ccc} E_{3} & = & E\{\underset{n\to \infty }{\text{lim}}n{\hat{\theta }}_{n}^{\left(3\right)}{\hat{\theta }}_{n}^{\prime (3)}I\left(L_{n}\leq l_{n,\alpha }\right)\},\;\text{by}\;\text{Theorem}\left(5\right)\\ & = & \pi \pi^{\prime }H_{p_{2}+4}\left(\chi_{p_{2}\left(\alpha \right)}^{2};\Delta \right)+B^{*}H_{p_{2}+2}\left(\chi_{p_{2}\left(\alpha \right)}^{2};\Delta \right). \end{array}$$

By combining *E*_1_, *E*_2_, *E*_3_, the results holds, and the $QR({\hat{\beta_{1}}}^{PTL})$ is obtained.

Similarly, we can proof parts (4) and (5), but we omit the proof to safe the space.

Analytical risk comparisons of the proposed estimators can be carried out based on *QR* expressions. However, our results are similar to those discussed by Al-Momani, M. *et al* [38] and Bahadir Y. *et. al* [36], so we relay on numerical comparisons to check the estimators performance.

## 6 Numerical study

In this section, we examine the performance of the proposed estimators numerically based on Monte Carlo Simulation experiments and real data example.

### 6.1 Monte Carlo Simulation

We conduct a Monte Carlo Simulation using square lattices of *N* × *N* with *N* = 7, 10 and corresponding sample sizes *n* = *N*^2^ = 49, 100. The design matrix ***X*** is generated from multivariate Gaussian distribution with mean (**0**) and covariance matrix with first-order Autoregressive structure for the assessment of multicollinearity. That is, $cov(X_{i},X_{j})=\{\begin{array}{cc} \rho_{X}^{\left|i-j\right|},\; & i\neq j\\ 1,\; & i=j \end{array}$, and use *ρ*_*x*_ = {0.3, 0.6, 0.9}. The error term ***ϵ*** is generated from multivariate Gaussian distribution with CA covariance structure, so ***ϵ*** ∼ *N*(0, *σ*^2^(***I***_*n*_−ρW*)^−1^***D***). We use *σ*^2^ = 1, and employed a queen-based contiguity neighborhood for the matrix W*. The spatial dependence parameter *ρ* is chosen to vary over the set {−0.9, −0.5, 0, 0.5, 0.90}. The *p* × 1 parameter vector ***β*** is partitioned as $\beta ={\left(\beta_{1},\beta_{2}\right)}^{\prime }=\left(1_{p_{1}},\Delta ,0_{p_{2}-1}\right)$, where $1_{p_{1}}$ is a *p*_1_ × 1 vector of ones, $0_{p_{2}-1\times 1}$ is a *p*_2_−1 vetoer of zeros, and Δ is the non-centrality parameter defined as Δ = ‖***β*** − ***β***_0_‖, where ‖.‖ is the Euclidian norm. The values of Δ are chosen to vary from 0 to 2. Obviously, when Δ = 0, the null hypothesis in (10) is true, and becomes false when Δ starts moving from the null space. The number of regression coefficients that form the vector ***β*** are (*p*_1_, *p*_2_) ∈ {(5, 10), (5, 20), (5, 30)}, and we use *α* = 0.05. To fit the full and sub CA models, we use the spdep R-package [39] and apply the function spautolm to the generated data. A 2000 Monte Carlo runs is repeated for each single case. In each of these runs, the full model, sub-model, pretest, shrinkage, and positive shrinkage Liu-type estimators of ***β***_1_ were computed, and the mean squared error (MSE) for all estimators obtained, then the simulated relative efficiencies(SRE) with respect to the full model MLE estimator ($\hat{\beta_{1}}$) of (*β*_1_) are calculated for all values of Δ using the following formula:

$$\begin{array}{ccc} SRE(\beta^{⋆}) & = & \frac{MSE(\hat{\beta_{1}})}{MSE(\left(\beta^{⋆}\right)}, \end{array}$$ (26)

where for any estimator for ***β***_1_, say $\beta_{1}^{◊}$, the $MSE(\beta_{1}^{◊})=\sum_{i=1}^{p_{1}}{\left(\beta_{1i}^{◊}-\beta_{1i}\right)}^{2}$, and ***β***^⋆^ is any of the estimators $\left\{\{{\hat{\beta_{1}}}^{LU},{\hat{\beta_{1}}}^{LUS},{\hat{\beta_{1}}}^{PTL},{\hat{\beta_{1}}}^{SL},{\hat{\beta_{1}}}^{PSL}\}\right\}$. If the SRE of ***β***^⋆^ is more than one, then it indicates superior to the full model Liu estimator, and vice versa. We noticed no significant difference exits when changing the spatial dependence parameter *ρ*, we only present the graphs for *ρ* = 0.90 for different values of Δ which appear below.

**Fig 1:**
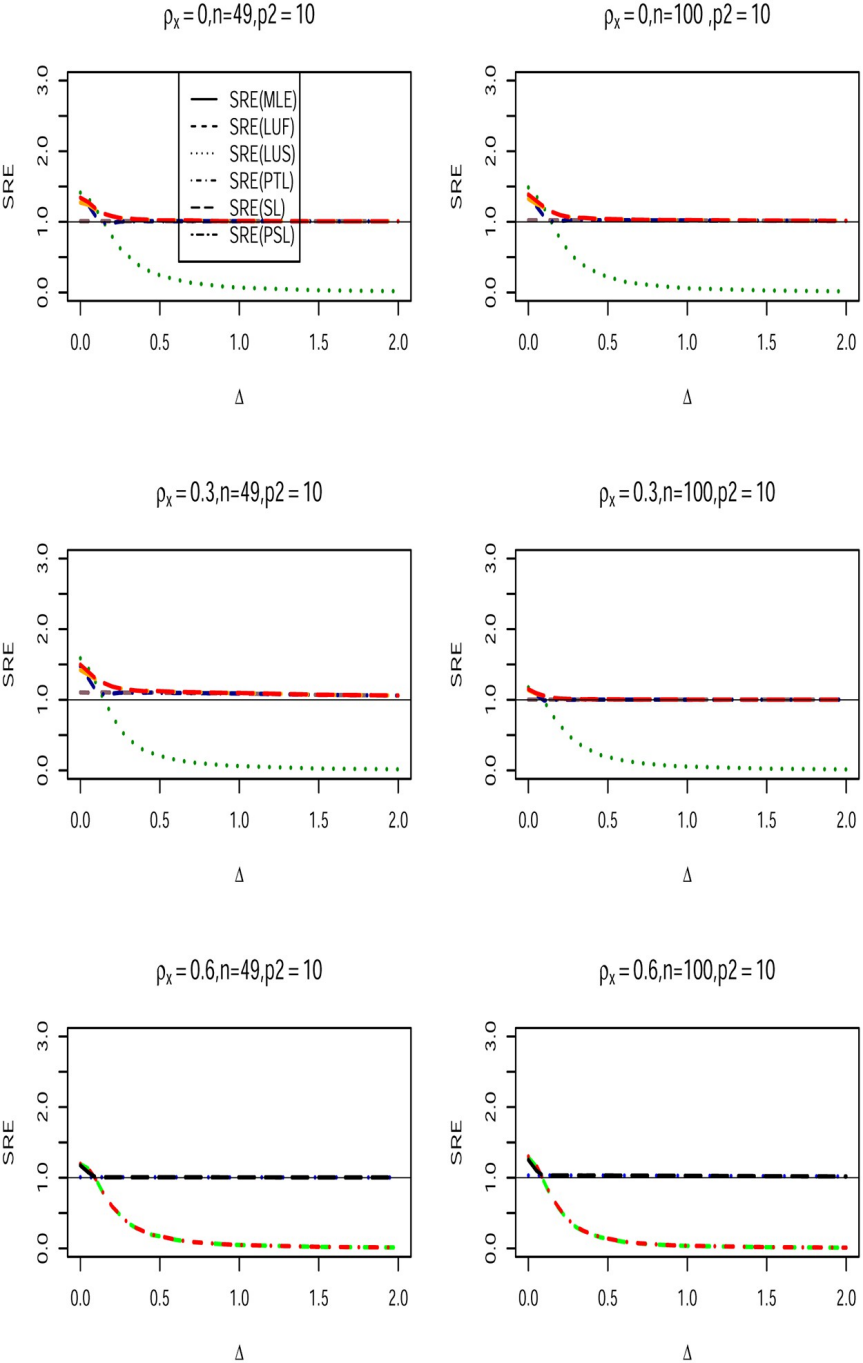
SRE of the proposed estimators with respect to (${\hat{\beta_{1}}}^{LU}$) when *n* = 49, 100, *ρ*_*x*_ ∈ {0.3, 0.6, 0.9}, *ρ* = 0.90, and (*p*_1_, *p*_2_) = (5, 10).

**Fig 2:**
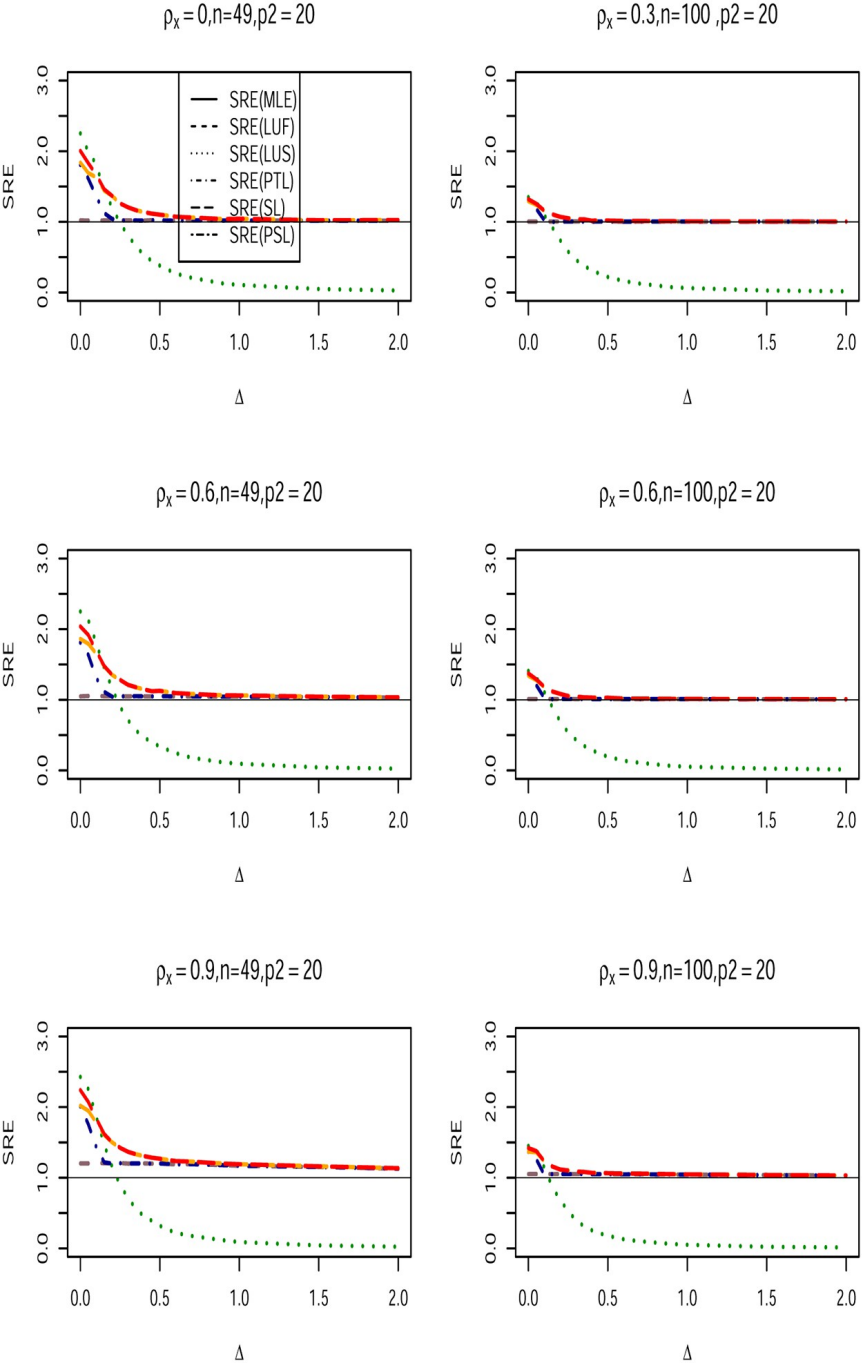
SRE of the proposed estimators with respect to (${\hat{\beta_{1}}}^{LU}$) when *n* = 49, 100, *ρ*_*x*_ ∈ {0.3, 0.6, 0.9}, *ρ* = 0.90, and (*p*_1_, *p*_2_) = (5, 20).

**Fig 3:**
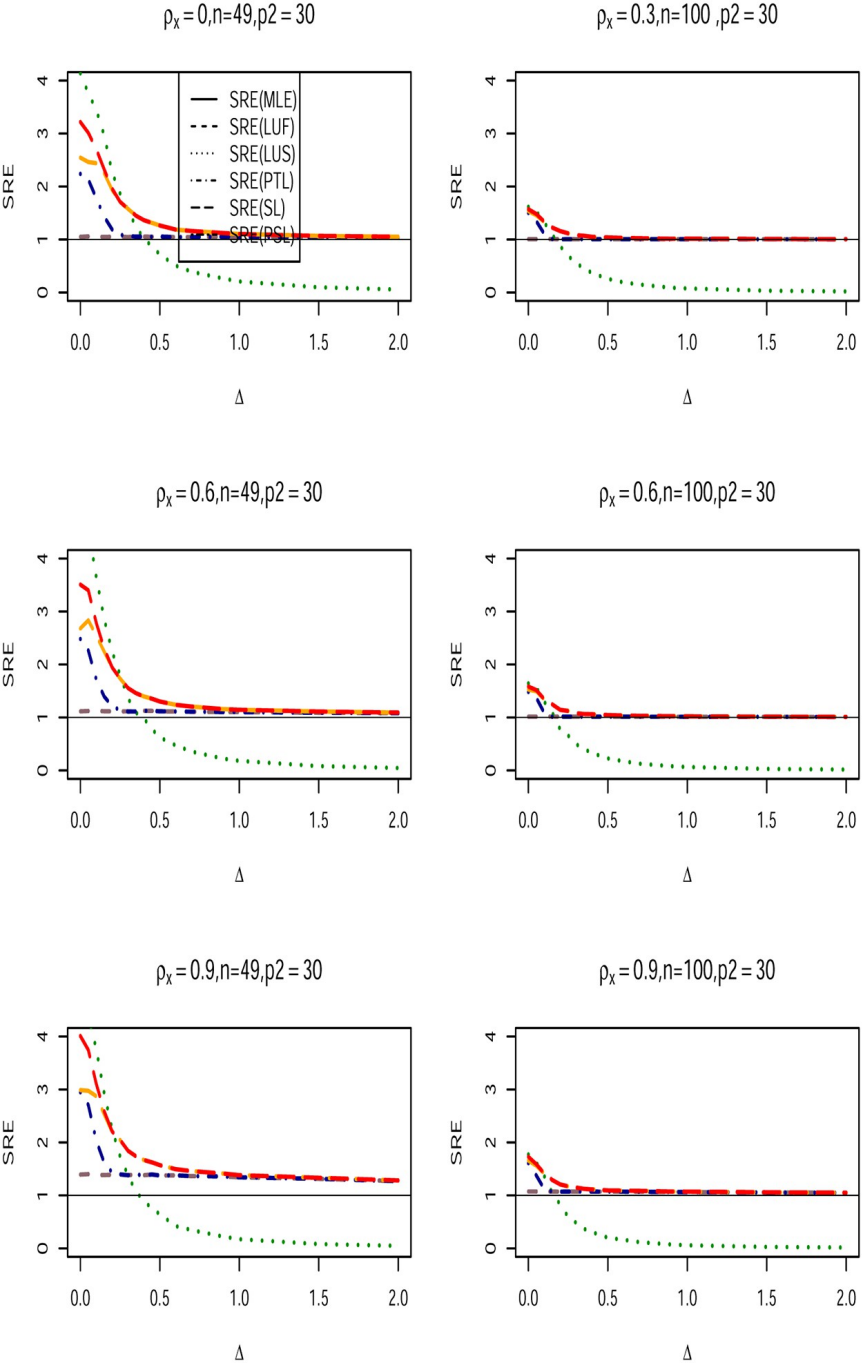
SRE of the proposed estimators with respect to (${\hat{\beta_{1}}}^{LU}$) when *n* = 49, 100, *ρ*_*x*_ ∈ {0.3, 0.6, 0.9}, *ρ* = 0.90, and (*p*_1_, *p*_2_) = (5, 30).

Figs (1)–(3) lead to the following conclusions:

1.
${\hat{\beta_{1}}}^{LU}$ dominates the classical MLE estimator uniformly over all values. Further, as *p*_2_ increases, its efficiency increases for fixed values of *ρ* and *ρ*_*x*_. Furthermore, ${\hat{\beta_{1}}}^{LU}$ efficiency increases as the multicollinearity becomes stronger among the explanatory variables within the design matrix.
2. When Δ = 0, the Liu-type sub-model estimator dominates all other estimators. It is expected as the null hypothesis is true. However, as Δ starts moving form the null space, the SRE of estimator decreases sharply, and the estimator becomes inefficient compared with rest of the estimators.
3. As the correlation coefficient *ρ*_*x*_ increase among the explanatory variables, the SRE values are also increase holding other parameters fixed.
4. The SRE of all estimators increase when the number of zero coefficients (*p*_2_) increases.
5. The Liu-type positive shrinkage estimator is uniformly dominates the other estimators.

### 6.2 Boston housing data

Regarding the use of housing market information for census tracts in the Boston Standard Metropolitan Statistical Area in 1970, Harrison and Rubinfeld [40] looked at a number of practical concerns. Their main goal was to determine the correlation between a group of (15) variables and the median price of owner-occupied homes in Boston. A corrected version of the data set with additional spatial information were provided by Gilly and Pace [41]. The data set is available under the R-Packages MASS, spdep, the list of the variables as given in the package are as follows:

- TRACT: Census tract id number.
- MEDV: Median value of owner-occupied homes in (1000’s USD).
- CMEDV: Corrected median values of owner-occupied housing in (1000’s USD).
- ZN: Proportions of residential land zoned for lots over 2500 square feet per town (constant for all Boston tracts).
- INDUS: Proportions of non-retail business areas per town.
- RM: Average numbers of rooms per dwelling.
- AGE: Proportions of owner-occupied units built prior to 1940.
- CHAS: A dummy variable with two levels, 1 if tract border to Charles river; 0 otherwise.
- NOX: Levels of nitrogen oxides concentration (parts per 10 million) per town.
- CRIM: Crime rate per capita.
- DIS: Weighted distance to five employment centers.
- RAD: An index of accessibility to radial highway per town (constant for all Boston tracts).
- LSTAT: Percentage of lower status population.
- TAX: Property tax rate per (USD 10,000) per town (constant for all Boston tracts).
- PTRATIO: Pupil-teacher ratios per town (constant for all Boston tracts).
- B: The variable *B* = 1000(*b* − 0.63)^2^, where b is the proportion of blacks.

**Fig 4:**
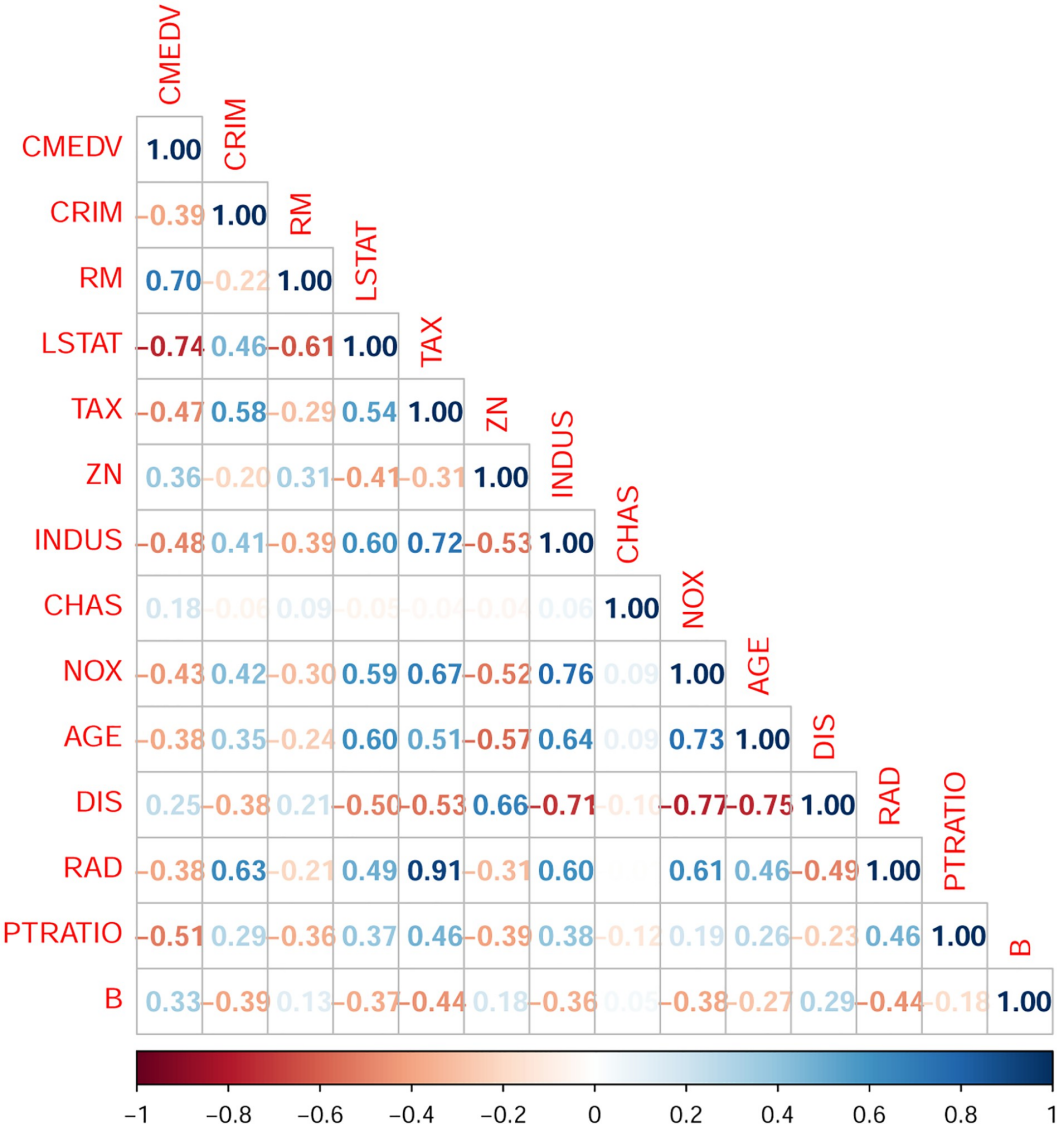
Correlation among all variables.

Fig (4) shows a plot of the correlation coefficients among all variables in colors in which the a strong linear relation appears in dark colors, and as it becomes weak, the color changes to light or may disappear when no linear relation exists. The figure shows some strong linear relationship between the CMEDV and some other variables. As we do not have any prior information about the available covariates, we might apply any variable selection method. In our scenario, we employ the AIC/BIC selection criterion to produce a submodel.

The full which contains all available covariates, and the sub model obtained by the AIC/BIC selection are given above in Table (1). To evaluate the performance of the proposed estimators, we used a bootstrapping method suggest by Solow [42], and computed the mean squared prediction error (MSPE) using each estimator as below:

1. We use the spautolm function to fit the CA full model using all available variables as papered in Table (1) and obtain the maximum likelihood estimates of ***β***, *σ*^2^, the spatial dependence parameter *ρ*, the matrix ***V***_*n*_ and the biasing parameter *d* using the formula suggested by Alheety *et. al* [43], which is given by:
  $$\begin{array}{ccc} d_{opt.} & = & 1-\frac{2p\sigma^{2}}{p\sigma^{2}+\beta^{\prime }\beta }. \end{array}$$ (27)
  and we estimate *d* and ***V***_*n*_ by replacing *σ*^2^, *ρ* and ***β*** by their corresponding MLEs estimates, where $\hat{\sigma^{2}}=\frac{\left(Y-X\hat{\beta }{)}^{\prime }(Y-X\hat{\beta }\right)}{n-p}$
2. Employ the Cholesky decomposition for the matrix $\hat{V_{n}}$ to write it as $\hat{V_{n}}=\hat{A}{\hat{A}}^{\prime }$, where $\hat{A}$ is an (*n* × *n*) lower triangular matrix.
3. Define the residual as $\hat{\epsilon }={\hat{A}}^{-1}(Y-X\hat{\beta })$, where $\hat{\epsilon }=(\hat{\epsilon_{1}},\hat{\epsilon_{2}},\ldots ,\hat{\epsilon_{n}})$ and define $\tilde{\epsilon_{i}}=\hat{\epsilon_{i}}-\frac{1}{n}\sum_{k=1}^{n}\hat{\epsilon_{k}},k=1,2,\ldots n$ as the centered residual.
4. Obtain a sample with replacement of size (*n*) from $\left(\tilde{\epsilon_{1}},\tilde{\epsilon_{2}},\ldots ,\tilde{\epsilon_{n}}\right)$ to get $\epsilon^{*}=(\epsilon_{1}^{*},\epsilon_{2}^{*},\ldots ,\epsilon_{n}^{*})$.
5. Compute the bootstrapping response value as $Y^{*}=X\hat{\beta }+\hat{A}\epsilon^{*}$.
6. Use the bootstrapped value Y* to fit both the full and sub models and obtain the values of the proposed estimators.
7. Compute the predicted value of the response variable using each estimator as $\hat{y_{ki}^{*}}=X_{1}\hat{\beta_{1}^{*}}+\hat{\rho^{*}}\sum_{j=1}^{n}W_{ij}^{*}(\hat{y_{kj}^{*}}-X_{j}\hat{\beta_{1}^{*}})$, where $\hat{\beta_{1}^{*}}$ is any of the estimators in the set $\left\{{\hat{\beta_{1}}}^{LU},{\hat{\beta_{1}}}^{LUS},{\hat{\beta_{1}}}^{PTL},{\hat{\beta_{1}}}^{SL},{\hat{\beta_{1}}}^{PSL}\right\}$.
8. Compute the square root of the MSPE for the k^*th*^ bootstrapping sample as
  $$\begin{array}{ccc} MSPE_{k}\left(\hat{\beta_{1}^{*}}\right) & = & \sqrt{\frac{\sum_{i=1}^{n}{\left(\hat{y_{i}^{*}}-y_{i}\right)}^{2}}{n}},k=1,2,\ldots ,K, \end{array}$$ (28)
  where *K* is the number of bootstrapping samples.
9. Compute the relative efficiency of the square root of the MSPE (REMSPE) as follows:
  $$\begin{array}{ccc} REMSPE(\hat{\beta_{1}^{{}^{\circ}}}) & = & \frac{MSPE({\hat{\beta_{1}}}^{LU})}{MSPE(\hat{\beta_{1}^{{}^{\circ}}})}, \end{array}$$ (29)
  where $\hat{\beta_{1}^{{}^{\circ}}}$ is any of the proposed estimators, and we use *K* = 2000 bootstrapping samples.

**Table 1:** Full and submodel for the Boston housing data.

Selection Criterion	Model
Full	log(CMEDV) = CRIME+I(RM^2)+log(LSTAT)+TAX+ZN+INDUS
	+CHAS+I(NOX^2)+AGE+log(DIS)+log(RAD)+PTRATIO+B
Submodel	log(CMEDV) = CRIME+I(RM^2)+log(LSTAT)+TAX

A value of the REMSPE grater than one indicates the superiority of the estimator in the denominator.

Table (2) above summarized the results of the relative efficiencies. The table indicates that the submodel estimator ${\hat{\beta_{1}}}^{SM}$ dominates all other estimators followed by the pretest estimator ${\hat{\beta_{1}}}^{PTL}$. This is expected if the chosen AIC/BIC model is accurate or roughly accurate. In addition, all proposed estimators of ***β***_1_ dominate the estimator ${\hat{\beta_{1}}}^{LU}$.

**Table 2:** REMSPE of the proposed estimators.

Estimator	*REMSPE*
${\hat{\beta_{1}}}^{SM}$	1.2693
${\hat{\beta_{1}}}^{PTL}$	1.2182
${\hat{\beta_{1}}}^{SL}$	1.1850
${\hat{\beta_{1}}}^{PSL}$	1.2044

## 7 Conclusion

In this paper, we proposed the pretest, shrinkage, and positive shrinkage estimators for the CA model’s large-scale effects vector of parameters. We formulated a hypothesis of the form *H*_0_ : ***β***_2_ = 0 to obtain the Liu estimator of the main effect ***β***_1_ under this UPI, and the submodel estimators. Then we combined these two estimators to get the Liu-type pretest, shrinkage and positive shrinkage estimators.

Further, the set of estimators were compared analytically based on their asymptotic bias, quadratic bias, and risks, and provided related expressions. Also, these estimators were evaluated numerically via their relative performance using an expensive simulation experiments based on different values of the spatial dependence parameter (*ρ*) and difference lattice sizes (*N*), and applied the proposed estimators to a real data example. Our analytical and numerical results showed that the submodel estimator is superior whenever the restriction given by *H*_0_ : ***β***_2_ = **0** is correct or nearly correct, that is when the UPI is true. However, when the restriction becomes false and the test statistics rejects the null hypothesis, the submodel estimator becomes inefficient, and had the highest MSE, while the Liu-type positive shrinkage estimator showed the highest performance compared with other estimators regardless of the accuracy of the UPI.

For future research, the proposed estimation approach might be applied to different spatial regression models and investigate the performance of the proposed estimators analytically and numerically. Also, one more attractive area is the extension of the proposed estimation strategies to the high-dimensional data (HDD) case of the large-scale effect regression parameter vector of the CA model when (*p* > > *n*), and study the behavior of the Liu-type estimators. In addition, we can study the Liu-type estimation technique assuming a prior distribution for the CA model, and obtain the updated Liu, pretest, shrinkage, and positive shrinkage Liu-type estimators of ***β***_1_.

## Supporting information

S1 Text (TXT)
